# Gut-Microbial Responses to Acute Polyester Microplastic Exposure in Zebrafish: Dysbiosis, Opportunistic Bacteria, and Functional Impact

**DOI:** 10.3390/ijms27125355

**Published:** 2026-06-13

**Authors:** Linus S. H. Lo, Liyuan Qiang, Peiyuan Ye, Cuizhu Ma, Keng Po Lai, Huahong Shi, Jinping Cheng

**Affiliations:** 1 Department of Science and Environmental Studies and State Key Laboratory of Marine Environmental Health, The Education University of Hong Kong, New Territories, Hong Kong, China; 2 College of Mechanical and Electrical Engineering, Shihezi University, Shihezi 832000, China; 3 State Key Laboratory of Estuarine and Coastal Research, East China Normal University, Shanghai 200241, China; 4 Department of Applied Science, School of Science and Technology, Hong Kong Metropolitan University, Hong Kong, China

**Keywords:** microplastics, gut microbiota, polyester, transcriptome, opportunistic bacteria

## Abstract

Microplastics are widespread environmental contaminants with adverse health impacts. The gastrointestinal tract represents a primary site for host–microplastic contact and interactions, but microplastic-driven perturbations of the gut microbiome and how they mediate toxicity to the gut and host’s health remain poorly elucidated. In this study, zebrafish (*Danio rerio*) were exposed to environmentally ubiquitous polyester microplastics and investigated for acute dysbiosis and host–microbiome molecular responses using an integrated histological and multi-omics approach. Gut transcriptomic results first revealed initial dysregulations under microplastic stress, increasing energy–metabolic activity and suppressing detoxification-associated pathways on day 3, followed by downregulated gut epithelial maintenance and anti-inflammatory responses by day 7. During this process, opportunistic bacterial taxa such as *Edwardsiella* and the microbial antioxidant biosynthesis pathway can be enriched transiently. The limited structural damage and modest microbiome alterations observed after acute exposure, however, may suggest partial resilience of the host gut and microbiome. This study demonstrates microplastic-induced gut impairment and host–microbiome responses to acute polyester microplastic stress, providing evidence to enable better characterization of the gut health risks associated with microplastic contamination.

## 1. Introduction

Despite growing environmental concerns, global annual plastic production has continued to surge from 370 million metric tons in 2018 to 430 million metric tons in 2024 [1], with the majority of plastics fated to accumulate persistently in landfills and the natural environment as pollutants [2]. Of particular concern are microplastics (MPs, <5 mm) derived from larger plastic debris. Their reduced size and increased abundance facilitate exposure risk via ingestion to a wider range of trophic levels [3], elevating their bioaccumulation and biomagnification potential and threat to environmental health exponentially. On a global scale, MP pollutants in the form of ocean litter have been evidenced in at least 1300 animal species [4]. Humans are also exposed to MPs, predominantly through daily dietary intake, where MPs originating from contaminated food, drinking water [5], beverage products [6], and related release from plastic containers such as bottles and cups [7] are inevitably ingested by humans. Accordingly, the various potential adverse effects of MP exposure have been frequently reviewed and investigated in the laboratory [8]. The reported negative health impacts include neurotoxicity [9], genotoxicity [10], reproductive toxicity [11], behavioral impacts [12], reduced growth and survival [13] and more. However, experimentally observed impacts were also highly variable across taxa and trophic levels [14], partially attributable to limited standardization across the wide range of exposure time and dosages used to invoke tipping points and worrying possibilities [15]. Many foundational findings are also inevitably built upon an incomplete account of host–MP interactions and associated mechanisms. As a result, multiple gaps in mechanistic understanding and molecular evidence remain before experimental observations can be bridged to more holistic and accurate environmental, ecological, and human health risk assessments [16]. This renders the extent of crucial health impacts induced by MP pollution an ongoing topic of debate in need of further empirical evidence and investigation.

In recent years, research investigating the disturbance to the gut by MPs has gained traction for the gut’s importance as a primary site of exposure and interaction, upstream to most adverse impacts. From our previous observation [17,18], in addition to growing global field evidence with fish [19,20,21], it was established that MP retention primarily occurs in the gut, followed by gills and other organs such as liver and brain, which MPs may translocate into [22]. The gut barrier thus represents the critical first line of defense. Under the continuous influx of MPs, fish have demonstrated a homeostatic ability to eliminate excessive MPs in a diel pattern relatively regularly [18], more so if allowed to depurate and recover in exposure-free isolation [23]. However, brief encounters with MPs can still cause abrasions and direct mechanical disturbance to the gut epithelium, linked to oxidative stress [24] and direct growth inhibition due to physical occlusion and food intake reduction [25]. In humans, the abundance of MPs observed in gut tissues, associated with their further biotranslocations to blood and organs [26], is reportedly linked to increased acute and chronic health conditions, such as inflammatory bowel disease and neurodegenerative disorders [27]. Understanding the gut’s response and underlying mechanisms that may mediate defense against stress or, alternatively, propagate toxicity to the gut and subsequently host health is therefore a critical gap in knowledge to be addressed.

Beyond physical impact, disturbances of the gut microbiota induced by MPs remain underexplored mechanisms mediating and regulating the manifestation of microplastic-induced toxicities. The gut microbiota plays a pivotal role in the bidirectional communication pathways with important brain [28], liver [29], and immune systems [30], typically referred to as the “gut microbiota–brain axis”. They provide signaling metabolites and neurotransmitters involved in the regulation of various host functions, including digestion, nutrient absorption, vitamin synthesis, immune regulation, and gut barrier maintenance [31,32]. MP exposure, by providing a substrate interface for microbial colonization and biofilm formation, can disturb microbial compositions and balance of commensal and pathobiont in the gut, referred to as gut microbiome dysbiosis [33,34]. Early evidence of gut microbiota disruptions caused by MP exposure has been linked with microbe-mediated inflammation, intestine permeability alterations [35], and oxidative stress [36]. Additionally, recent studies showed that MPs can selectively enrich pathogens on their surface biofilms [37]. However, the capacity and to what extent various MP exposure may harmfully alter gut microbiota, influence functionality associated with host health and defense, and induce health risks are still not fully characterized. The potential responses by the gut microbiota to mediate stressful encounters have also been sparsely explored to date. A more thorough investigation of MP exposure in the gut, the potential microbial adaptation and selection induced on the gut microbiota’s functional composition, and how they may enable reported neurological, developmental, and health disorders will contribute to an improved ability to accurately assess the multifaceted risks presented to the environment and humans.

In this study, we employed the zebrafish model and a combination of gut transcriptomic and 16S rRNA metabarcoding-based functional analyses to dissect the acute impacts of gut MP exposure on gut microbiota composition and associated stress responses. Approximately 70% of human genes have a zebrafish orthologue [38], rendering zebrafish a valuable model for their homology with vertebrates and mammals to study key metabolic, immune, and gut–brain axis pathways [39,40]. Accordingly, we hypothesize elevated MP exposure to the gut continually poses potential compound health risks in the forms of mechanical burden and stress to energy and host defense-related functions of the gut. MP exposure may alter the abundances of opportunistic bacterial taxa, while some microbial functions associated with stress responses may also become enriched. The aims of this study were two-fold: (1) to characterize MP-induced acute stress responses in the gut and (2) to explore potential MP-induced structural and functional dysbiosis of the gut microbiota. We place a special focus on revealing the potential increase in opportunistic bacteria among the gut microbiota composition to reflect potential health risks exploiting host vulnerability under continual MP exposure. The findings of this study will expand our knowledge of the stress responses of high-trophic-level consumers to MP exposure in the gut. This has implications for host stress tolerance, fitness, and competence for survival, thereby enabling a more thorough assessment of the practically inevitable health and ecological risks imposed by MP pollution on aquatic organisms and humans.

## 2. Results

### 2.1. MP Exposure in the Gut Did Not Reduce Intestinal Mucus Secretion

**Figure 1:**
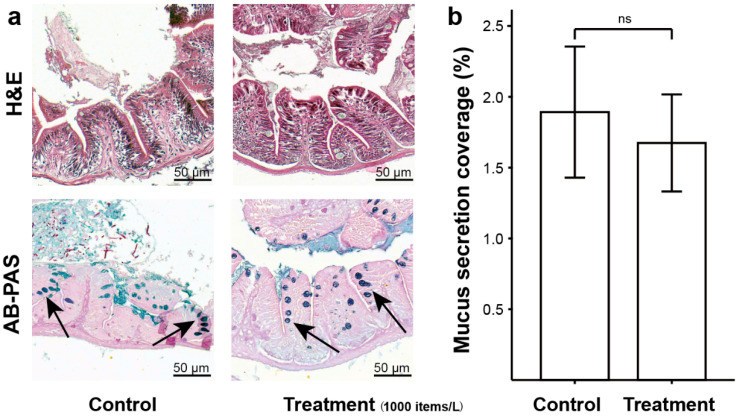
Representative transverse sections of zebrafish gut and quantification of mucus secretion after 7 days of microplastic exposure at 1000 items/L. (**a**) Hematoxylin and eosin (H&E) staining and Alcian Blue-Periodic Acid Schiff (AB-PAS) staining of zebrafish gut tissue from the treatment and the control groups. Images were captured at 80× magnification, with arrows indicating mucus compartments within the gut. (**b**) Quantification of mucus secretion in the zebrafish gut. The “ns” mark denotes no statistically significant differences between the treatment and control groups (independent *t*-test, *p* > 0.05).

Histopathological analysis of the zebrafish intestinal structure was first conducted to assess the direct and mechanical effects of MP exposure prior to subsequent transcriptomic and gut microbiota analyses. The staining results from tissue sections showed that, after 7 days of MP exposure, the zebrafish intestinal structure remained largely intact (Figure 1a and Figure S1). The intestinal epithelium remained continuous and well-organized, with no observable disruption or detachment, while the villi retained their overall morphology, showing no evidence of atrophy or structural deformation. Quantitative analysis of the mucus secretion coverage ratio revealed a decrease in the treatment group (1.67%) compared with the control group (1.89%); however, the difference was not statistically significant (independent *t*-test, *p* > 0.05; Figure 1b).

### 2.2. Initial MP Exposure Induced Increased Energy Demand and Suppressed Detoxification in Zebrafish Gut

**Figure 2:**
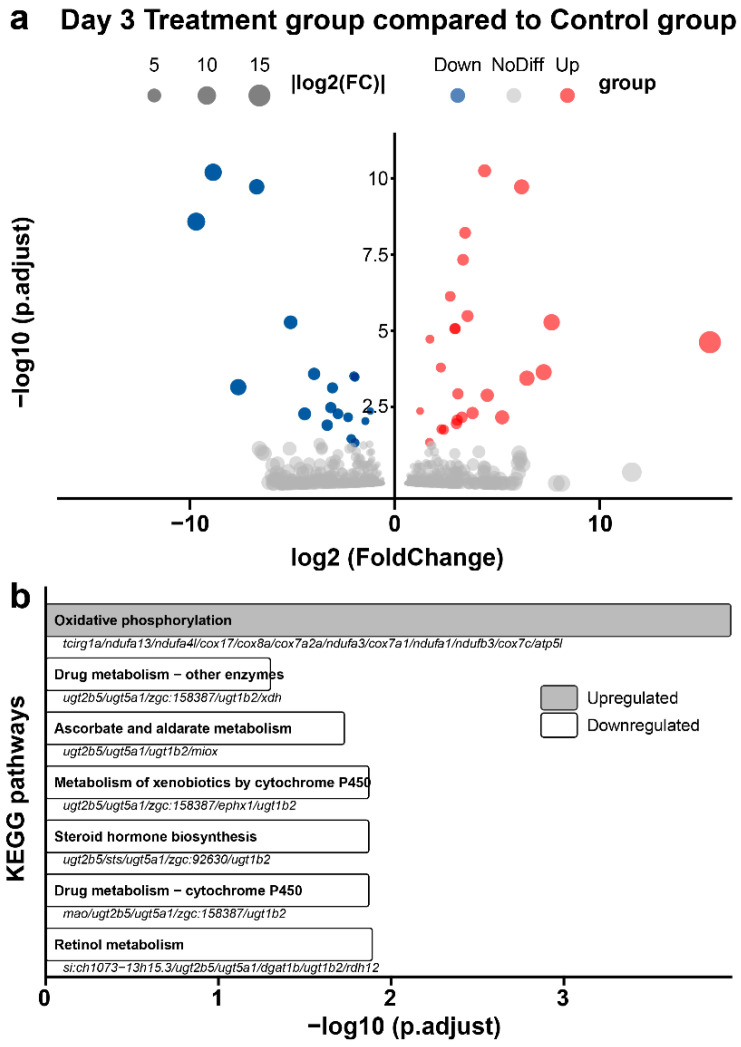
Dysregulated gene expressions involved in energy production, metabolism, and detoxification responses in the zebrafish gut after 3 days of microplastic exposure at 1000 items/L. (**a**) Volcano plot showing differentially expressed genes (DEGs) in zebrafish gut tissue between the treatment and control groups. Blue-colored circles indicate downregulated genes, and red-colored circles indicate upregulated genes. Grey-colored circles indicate genes without significant difference. (**b**) KEGG pathways enriched and suppressed by upregulated and downregulated DEGs on day 3. All significantly enriched and suppressed pathways are shown, and differentially expressed genes within these pathways are displayed below the bars. P.adjust represents the Benjamini–Hochberg-adjusted *p*-value controlling the false discovery rate ≤ 0.05. FC: Fold Change. KEGG: Kyoto Encyclopedia of Genes and Genomes.

Differentially expressed genes upon MP treatment were investigated to assess the impact, potential mechanisms, and functional changes in the fish gut, with a focus on hypothesized energy production and defense-related processes. A comparative analysis between the control and 3-day MP treatment groups identified 43 differentially expressed genes (DEGs), including 25 upregulated and 18 downregulated genes (Figure 2a). Given the coordinated nature of transcriptomic regulation, pathway-level enrichment was prioritized to reduce over-interpretation of isolated gene-level changes.

To understand the functional pathways affected by MP exposure, the DEGs were subjected to Kyoto Encyclopedia of Genes and Genomes (KEGG) and Gene Ontology (GO) pathway enrichment analyses. KEGG pathway annotation (Figure 2b) first showed significant upregulation of the oxidative phosphorylation pathway, driven by 12 upregulated oxidative phosphorylation genes, suggesting increased energy production and metabolic activity in the gut. Downregulated pathways include drug metabolism, metabolism of xenobiotics by cytochrome P450, and drug metabolism—cytochrome P450, indicating reduced ability of the gut microbiota to metabolize and detoxify xenobiotics and drugs; ascorbate and aldarate metabolism; steroid hormone biosynthesis; and retinol metabolism, which drive the metabolism of vitamins and hormones. Alternatively, GO analysis annotations revealed that 3-day MP exposure induced upregulation of genes that broadly belonged to 22 terms, eight of which fall under mitochondria-associated cellular components and 14 under molecular functions, while downregulated genes belonged to 14 terms, including oxidative-reduction processes, extracellular region-associated, and another 11 molecular functions mostly related to peptidase regulator and inhibitor activity (Figure S2).

### 2.3. Acute MP Exposure May Undermine Gut Epithelium Maintenance and Lipid Metabolism in Fish Gut

**Figure 3:**
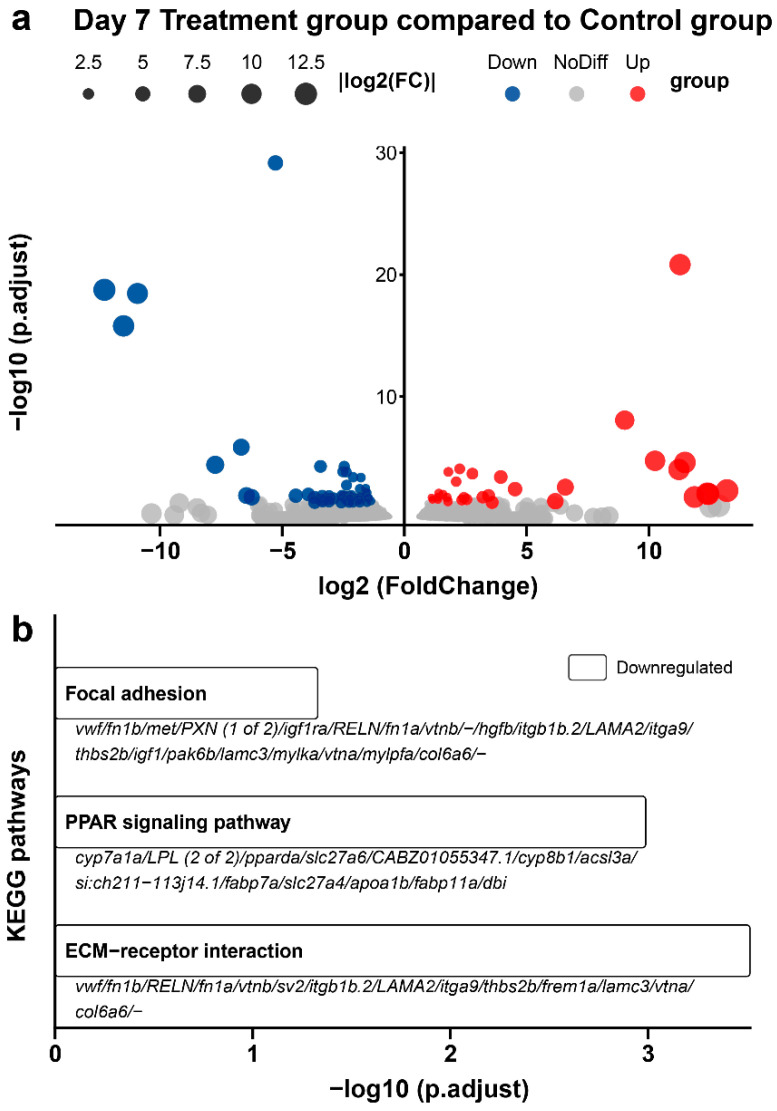
Dysregulated gene expressions involved in gut epithelium maintenance and lipid metabolism in the zebrafish gut after 7 days of microplastic exposure at 1000 items/L. (**a**) Volcano plot showing differentially expressed genes (DEGs) in zebrafish gut tissue between the treatment and control groups. Blue-colored circles indicate downregulated genes, and red-colored circles indicate upregulated genes. Grey-colored circles indicate genes without significant difference. (**b**) KEGG pathways enriched and suppressed by upregulated and downregulated DEGs on day 3. All significantly enriched and suppressed pathways are shown, and differentially expressed genes within these pathways are displayed below the bars. P.adjust represents the Benjamini–Hochberg-adjusted *p*-value controlling the false discovery rate ≤ 0.05. FC: Fold Change. KEGG: Kyoto Encyclopedia of Genes and Genomes.

To further investigate the extent of gene dysregulation in the fish gut under continued MP exposure, a similar comparative transcriptomic analysis was conducted, identifying a total of 86 DEGs, including 31 upregulated and 55 downregulated genes (Figure 3a).

From the KEGG pathway analysis (Figure 3b), the downregulation of focal adhesion and extracellular matrix (ECM)-receptor interaction pathways were observed, which may indicate reduced cell adhesion, signaling, tissue repair and integrity. Further downregulation of the peroxisome proliferator-activated receptor (PPAR) signaling pathway is also associated with reduced lipid metabolism and anti-inflammatory responses, which may indicate a reduced ability to metabolize lipids and carry out functions that help modulate host inflammation or maintain lipid homeostasis and metabolic balance. As these pathways contribute to gut structural integrity and metabolic regulation, their downregulation may subtly contribute to weakening of gut barrier function, nutrient absorption, inflammation responses, and overall gut health. Alternatively, GO analysis annotation (Figure S3) revealed upregulated genes belonging to five terms in lipid and macromolecule transport and localization. Downregulated genes belonged to a wider range of 32 terms but remained consistently comprised of genes involved in the oxidation-reduction process, extracellular proteins, and various (endo)peptidase and enzyme inhibitor and regulator activity.

### 2.4. Zebrafish Gut Microbiota and Opportunistic Bacteria Perturbations Under MP Stress

**Figure 4:**
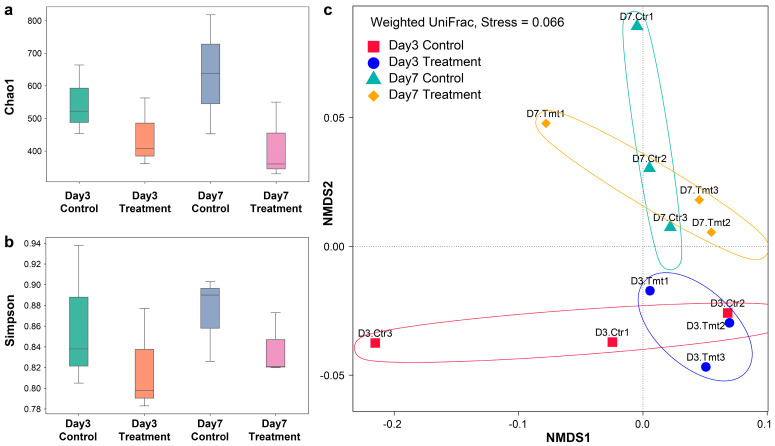
Changes in zebrafish gut microbiota alpha and beta diversity over time under microplastic exposure. Box plots showing the (**a**) Chao1 index for species richness and (**b**) Simpson index for microbial diversity and evenness among the control and microplastic-treated groups. (**c**) Non-metric multidimensional scaling (NMDS) plots showing differences in microbial community among all samples based on weighted UniFrac distances.

Analysis of gut microbiota alpha diversity revealed that MP exposure reduced microbial richness and diversity in zebrafish intestines. The Chao1 index, which reflects community richness, was consistently lower in the treatment group than in the controls at both time points. On day 3, the Chao1 index decreased from approximately 547 in controls to 444 in treatments, while on day 7, it dropped from 636 in controls to 414 in treatments (Figure 4a). The Simpson index also highlighted consistent declines in microbial diversity and evenness for the MP-exposed treatment groups from a mean of 0.86 in controls to 0.82 in treatments on day 3 and from 0.87 to 0.84 on day 7 (Figure 4b). However, the changes were not statistically significant. Similarly, non-metric multidimensional scaling (NMDS) ordination analysis revealed an overall stronger impact of temporal change on the distinct patterns in the gut microbial community structure when compared to MP exposure. When samples were grouped by exposure days, the weighted UniFrac analysis (Figure 4c) first showed clusters with limited dissimilarity between the treatment and control groups as compared to the day 3 and 7 groups. This showed that samples have retained relatively similar compositional structure in terms of relative abundances of top dominant taxa between the treatment and control groups (adonis test, *p* > 0.05).

**Figure 5:**
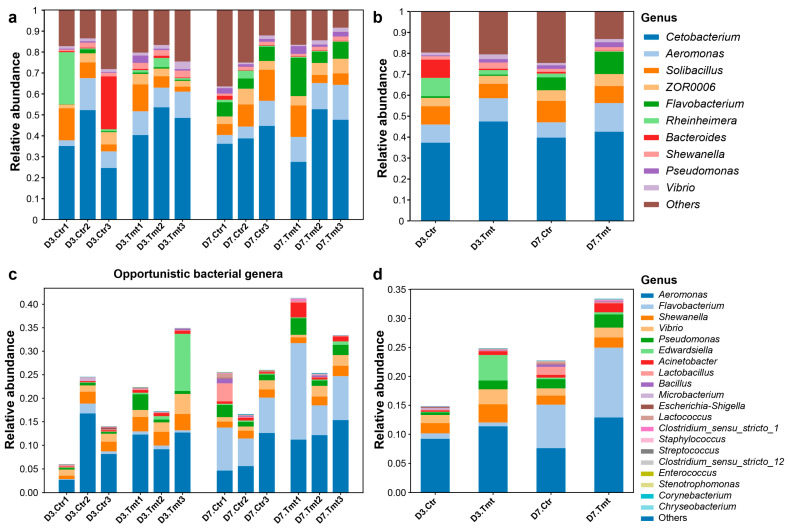
Changes in zebrafish gut microbiota composition and opportunistic bacterial abundances. Stacked bar charts illustrating the gut microbiota composition at the genus level in control and microplastic-treated groups after 3 and 7 days of microplastics exposure; data are presented either by (**a**) individual samples or grouped by (**b**) treatment conditions and the relative abundance of aquatic animal-related opportunistic bacterial taxa screened at the genus level across (**c**) different samples and (**d**) mean abundance in each group. D3: Day 3; D7: Day 7; Ctr: Control; Tmt: Treatment.

Following the NMDS analysis, the microbial community structure of the gut microbiota was analyzed at the genus level to examine the changes in both dominant and rarer bacterial taxa as well as the occurrence of opportunistic bacterial taxa after MP exposure. *Cetobacterium* was the most dominant genus observed in fish gut microbiota, followed by *Aeromonas* (10.2%), *Solibacillus* (8.5%) and *ZOR0006* (4.7%), which were present at moderate relative abundances across all groups, showing only minor variations between the control and treatment groups (Figure 5a,b). While the members of dominant bacterial taxa remained stable throughout the study period, some increases in relative abundance for notable genera containing known opportunistic bacteria species following MP exposure were also shown, including, but not limited to, *Aeromonas*, *Shewanella* and *Vibrio* (Figure 5c,d).

**Figure 6:**
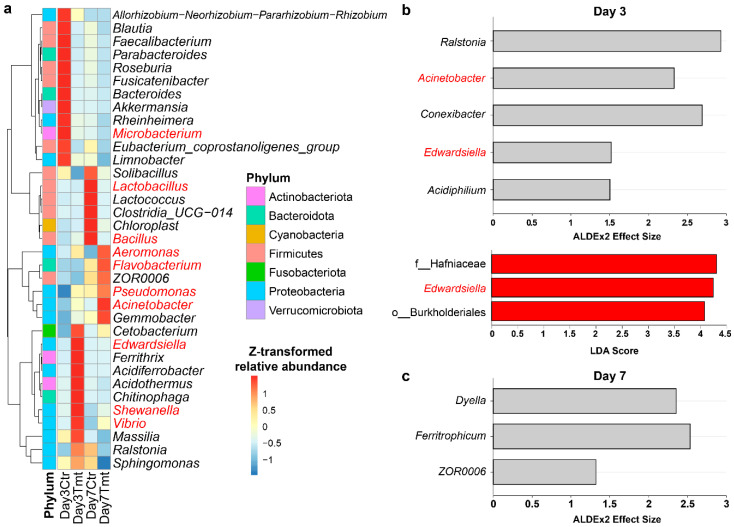
Differential abundance analysis of zebrafish gut microbiota between control and 1000 items/L microplastic treatment groups. (**a**) Heatmap showing genus-level differences in gut microbiota composition. Relative abundances are z-score-transformed. (**b**) ALDEx2 and linear discriminant analysis (LDA) effect size (LEfSe) analyses showing the effective size and linear discriminant analysis (LDA) score of identified differentially abundant taxa between day 3 and (**c**) day 7 treatment and control groups. Genera containing potential opportunistic bacteria are highlighted in red. D3: Day 3; D7: Day 7; Ctr: Control; Tmt: Treatment.

A hierarchical clustering heatmap was first used to visualize the potent bacterial compositional changes at the genus level that may contribute to gut microbiota dysbiosis (Figure 6a). The results showed that MP treatment significantly affected Proteobacteria genera from days 3 to 7, and notable opportunistic bacterial taxa, such as *Acinetobacter*, showed a significantly higher abundance in the treatment group (0.65%) compared to the control group (0.24%; *p* < 0.05). Conversely, on day 7, *Lactobacillus* showed a significant decrease in the treatment group (0.16%) compared with the control group (1.36%; *p* < 0.05), suggesting a disrupted commensal bacterial composition. Differentially abundant taxa were then verified using ALDEx2 and linear discriminant analysis (LDA) effect size (LEfSe) analyses. The results identified five tentative differentially abundant genera in *Ralstonia*, *Acinetobacter*, *Conexibacter*, *Edwardsiella* and *Acidiphilium* in the treatment group of day 3 as compared to the control (Figure 6b). This is aligned with LEfSe results, which identified *Edwardsiella* corresponding family Hafniaceae to be the only significant differentially abundant taxa between the two groups. The analyses were then repeated on day 7 samples, of which ALDEx2 results showed that *Dyella*, *Ferritrophicum*, and *ZOR0006* were differentially abundant taxa. However, these findings were not supported by LEfSe results and are conservatively omitted.

### 2.5. Microbial Antioxidant Defense Against Initial MP Exposure

**Figure 7:**
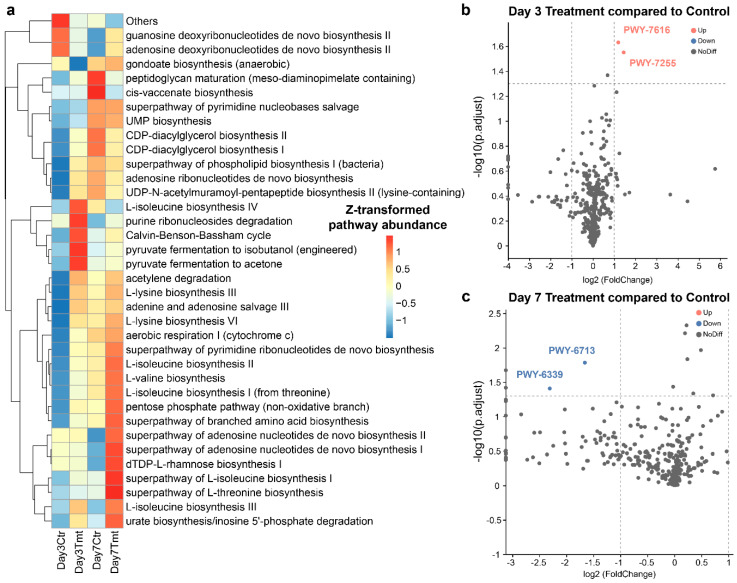
Differential abundance analysis of zebrafish gut microbiota functional potential between control and 1000 items/L microplastic treatment groups. (**a**) Heatmap showing pathway abundance differences of gut microbiota subjected to microplastic treatment. Top 35 pathway abundances shown are z-score-transformed. (**b**) Volcano plot showing differentially abundant pathways in zebrafish microbiota between the treatment and control groups on day 3 and (**c**) day 7. Dashed lines indicate the fold change threshold of |log2FoldChange| > 1 and an adjusted *p*-value threshold of ≤ 0.05. D3: Day 3; D7: Day 7; Ctr: Control; Tmt: Treatment.

Predicted microbial functional pathways were first overviewed to reveal potential shifts induced by MP exposure. The top 35 pathways are summarized in Figure 7a, which are mostly related to general microbial growth and core metabolism as expected. Differential abundance analysis on day 3 (Figure 7b) showed that PWY-7255 corresponding to ergothioneine biosynthesis and PWY-7616 corresponding to methanol oxidation to carbon dioxide were the only two significantly upregulated pathways. Increased ergothioneine biosynthesis may reflect selection for microbial antioxidant functions under a more oxidative or stressful gut environment. In contrast, increased methanol oxidation likely reflects a shift in community metabolic composition rather than a marker of gut damage. Similarly, differential abundance analysis on day 7 (Figure 7c) revealed downregulated PWY-6713 and PWY-6339 corresponding to L-rhamnose degradation II and syringate degradation, respectively, which are mostly reductions to plant/lignin sugar utilization by microbes less relevant to microbial defense.

## 3. Discussion

### 3.1. Acute PES-MP Exposure Induces Initial Stress Responses Dysregulating Metabolic Processes in the Gut of Zebrafish

On day 3, MP exposure first induced zebrafish gut transcriptional changes, with notable dysregulation of genes involved in energy production and detoxification responses. The early response of the zebrafish gut transcriptome reveals a robust upregulation of the oxidative phosphorylation pathway, evidenced by increased expression of key mitochondrial genes, including *tcirg1a*, *ndufa13*, *ndufa4l*, *cox17*, *cox8a*, *cox7a2a*, *ndufa3*, *cox7a1*, *ndufa1*, *ndufb3*, *cox7c*, and *atp5l*. These genes encode subunits of electron transport chain complexes I, IV, and V, which are responsible for ATP generation via aerobic respiration [41]. This upregulation may indicate that gut epithelial cells are mounting an energetic response to MP-induced stress. The metabolic shift is a well-documented acute stress response to supply protective mechanisms [42], such as cell proliferation, migration, and the synthesis of stress proteins. Concurrently, the transcriptome also demonstrated significant downregulation in several pathways critical for the gut’s defense and homeostasis. Key DEGs include ugt family genes *ugt2b5*, *ugt5a1*, and *ugt1b2*, which are involved in multiple downregulated metabolic pathways.

First, together with repressed genes such as *rdh12* and *dgat1b*, retinol metabolism and the processing of vitamin A are reduced. Retinol and its metabolites are essential regulators for the intestinal barrier function, production of mucous layer [43], and epithelial differentiation [44]; their suppression could predispose the gut to impaired regeneration and increased susceptibility to opportunistic bacteria. Reduced expression of *miox* and ugt family genes involved in ascorbate and aldarate metabolism also indicates impaired vitamin C metabolism and recycling. Since vitamin C is a key antioxidant, this downregulation may further compromise the tissue’s ability to neutralize reactive oxygen species that are likely generated during heightened oxidative phosphorylation [45]. Downregulation of *mao*, *zgc:158387*, *ephx1*, and *xdh* in drug and xenobiotic metabolism pathways suggests compromised detoxification capacity. The gut’s ability to neutralize MP-associated chemicals and other environmental toxicants and compounds may be reduced under prolonged retention, increasing the risk of toxin accumulation and cellular injury [46]. In conjunction with lower expression of *sts* and *zgc:92630*, suppressed steroid hormone biosynthesis may disrupt local hormone synthesis, thereby affecting immune regulation, stress responses, and tissue repair. Taken together, the combined upregulation of energy production and downregulation of metabolic pathways displayed early signs of an MP-imposed stress response, where increased ATP production is prioritized at the expense of long-term maintenance mechanisms, such as detoxification, antioxidant defense, and hormonal balance. Disrupted steroid and xenobiotic metabolism may also alter immune cell recruitment and inflammatory signaling within the gut, potentially increasing the vulnerability of the host. Interestingly, these results coincided with the upregulated microbial pathway for ergothioneine biosynthesis. Ergothioneine is a natural antioxidant synthesized by actinobacteria with anti-inflammatory and cytoprotective properties [47]. Under a stressful environment, the gut microbiota may be selected for increased microbial antioxidant functional potential, but whether this can contribute as a form of microbe-assisted defense for the host remains a functional hypothesis to be further investigated.

### 3.2. Reduced Gut Epithelial Structure-Related Gene Expressions Induced by Continuous MP Exposure

The results from this study showed that continued exposure to polyester (PES) MPs reinforced a more widespread downregulation of structural and metabolic pathways. From the KEGG pathway analysis, there is no evident continuation of responses observed in day 3 or upregulation of protective pathways to accompany the downregulation of focal adhesion and ECM–receptor interaction pathways observed. Genes governing tissue structure, cell–matrix interaction, and regeneration in the focal adhesion and ECM–receptor interaction pathways were extensively downregulated. These genes encode ECM components and ECM-binding cell-surface receptors, such as integrin subunits (*itgb1b.2*, *itga9*), fibronectin (*fn1a*, *fn1b*), laminin (*LAMA2*, *lamc3*), collagen VI (*col6a6*), and vitronectin (*vtnb*, *vtna*) as well as cytoskeletal and signaling proteins that mediate cell adhesion, migration, and survival signaling. A reduced capacity of the gut epithelium to adhere tightly to the ECM can compromise not only the physical barrier and permeability that separate the host from the external environment but also its recovery due to weakened signaling that promotes cell survival, leading to apoptosis or anoikis [48]. However, significant structural changes were not observed in this study. The substantial downregulation of the PPAR signaling pathway and related metabolic genes can also lead to the collapse of lipid metabolism and energy homeostasis. These genes are central to fatty acid uptake and intracellular transport (*slc27* family, *fabps*), triglyceride breakdown and fatty acid utilization (*LPL*, *acsl3a*), bile acid synthesis and cholesterol catabolism (*cyp7a1a*, *cyp8b1*), lipid-responsive transcriptional regulation (*pparda*), and lipoprotein assembly and lipid transport (*apoa1b*). Suppression of these pathways may constrain the gut’s ability to efficiently absorb and utilize lipids as an energy source [49] and for membrane structure and signaling, which, by extension, may lead to cellular stress and impair the function of both enterocytes and immune cells. PPARs are also anti-inflammatory transcription factors [50], and their reduced expression may, over time, contribute to chronic inflammation and compounding tissue damage if the exposure prolongs, but this will require further validation.

Overall, transcriptomic results on days 3 and 7 suggested that the fish gut under PES-MP stress has reduced gene expressions related to epithelial anchorage and tissue structural support. The downregulation of focal adhesion and ECM–receptor signaling undermines tissue cohesion and repair. Downregulated PPAR signaling and lipid metabolism may disrupt the gut’s ability to meet its energetic, nutritional, and immunodefense needs. However, both longer term studies and proteomics-based confirmation will be needed to verify this trajectory of a persistent, worsening barrier dysfunction from earlier suppression of antioxidant and detoxification pathways. These transcriptional changes may potentially contribute to altered inflammatory and metabolic responses during prolonged exposure.

### 3.3. Dynamic Gut Microbiota Perturbations: Commensals and Increased Opportunistic Bacteria Under Stress

The gut microbiota is closely associated with the health status of the host. Previous studies have evidenced that MPs acted as an aquatic pollutant to affect gut microbiota of aquatic animals [51], leading to gut oxidative stress and intestinal inflammation in fish [52]. In zebrafish studies, disturbed gut microbiota can reportedly significantly affect fish intestinal epithelial differentiation [53], epithelial cell proliferation [54], regulation of intestinal mucus changes [55], nutrient metabolism [56], immune responses [57], and brain health via the microbiota–gut–brain axis [58]. However, the current study revealed that, although acute exposure to PES-MPs decreased gut microbial diversity and evenness, the change was not statistically significant, contrasting with previous findings in guppies [59]. Dominant taxa of the gut microbiota detected in this study were *Cetobacterium*, a typical member of the gut core microbiota [60], and *Aeromonas*, which has known roles as both an opportunistic pathobiont [61] and signaling the regeneration of intestinal epithelial cells [35]. Based on recent studies demonstrating MPs as carriers and selective substrates for pathogenic bacteria [37,62,63], MPs are pollutants inferred with plausible capacities to subtly enrich opportunistic pathogen and pathobionts in the gut, with implications for elevated opportunistic infection risks when their abundance is unbalanced among the gut microbiome, coupled with MP-triggered immune impairments, inflammations, and a vulnerable host. This capacity has, however, only been limitedly investigated in the context of gut environments.

From our screening results on day 3, we showed that, despite the generally low abundance of opportunistic bacteria within the gut microbiota, PES-MP exposure can lead to a potential significant increase in the relative abundance of *Acinetobacter* and *Edwardsiella* in the treatment group compared to the control group (ALDEx2 effect size > 1.5). *Acinetobacter* are bacteria sporadically found in MP biofilms [37], and certain species produce pro-inflammatory cytokines closely associated with intestinal inflammation [64], while Edwardsiellosis is one of most critical bacterial diseases in fish causing enteritis and scepticemia [65]. A recent study by Geng et al. [66] showed that, in *Edwardsiella piscicida* infections, the depletion of intestinal T cells can cause sharp declines in *Cetobacterium* accompanied by an increase in opportunistic bacteria such as *Klebsiella* and *Acinetobacter* in the fish gut. This observation may concur with current evidence. However, current results also showed that, even at above environmental MP concentrations, zebrafish gut microbiota can generally cope with inferred MP and oxidative stress, as *Cetobacterium* showed no significant decline and enriched opportunistic bacterial abundance did not persist beyond day 7. In contrast, on day 7, the abundance of *Lactobacillus* was observed to significantly decrease in the treatment group compared to the control group. Previous studies have demonstrated that *Lactobacillus* can alleviate intestinal damage through various mechanisms and is better recognized as a commensal than its pathogenic potential. These functions include regulating the proportion of immune cells (e.g., *Treg/Th17*), secreting anti-inflammatory factors, enhancing tight junctions of intestinal epithelial cells, and maintaining the integrity of the mucous layer through modulating Wnt signaling pathways [67], thereby effectively improving the gut’s epithelial, mucous, and immune barriers [68].

Overall, the results showed that MP exposure may influence several gut-associated molecular pathways and cause compositional perturbations to the gut microbiota even in the absence of substantial physical injury. While we found limited microbial dysbiosis and an increase in opportunistic bacterial abundances, we emphasize that, compared to controlled experiments, environmental MPs are only more likely to harbor more complex plastisphere biofilms and diverse environmental pathogens. In light of predicted increasing environmental MP concentrations, urbanized water bodies have been increasingly reported as MP hotspots, with some of the localized concentrations, such as those in Baotou sections of the Yellow River, already exceeding treatment concentrations employed in laboratories and this study (>2500 items/L) [69]. Without mitigation strategies, the environmental bioaccumulation burden is likely to be exacerbated onto higher trophic level consumers [70], rendering the potential health and toxicity risk observed in the laboratory increasingly relevant. This has multiple implications for humans stemming from established widespread contamination of drinking water systems [71], continuously intensifying seafood and bivalve production for consumption [72,73], lack of efficient replacement for food-related products such as tea bags and packaging [74], and more, continuously perpetuating existing MP exposure risks. Considering the inherent limitations of this study, such as a limited sample size, exposure duration, and the resolution of metabarcoding-based screening and functional profiling, a significant amount of research is still required to elucidate the long-term ecological and public health risks associated with MP contamination. Future studies should incorporate downstream functional assays, metatranscriptomics, and metabolomics to validate mechanisms and improve our ability to characterize microplastic-associated risks.

## 4. Materials and Methods

### 4.1. Fish Maintenance and Microplastic Exposure

A total of 72 adult zebrafish (*Danio rerio*, AB strain) with body lengths of 3–4 cm were purchased from Shanghai FishBio Co., Ltd. (Shanghai, China). The zebrafish were randomly assigned to two groups: 36 zebrafish were designated for the experimental group, and 36 zebrafish were assigned to the control group. Prior to the experiment, all zebrafish were acclimated in a laboratory aquaria for at least two weeks to stabilize their physiological conditions. During this acclimation period, the aquarium conditions were maintained at 25 ± 1 °C, pH 7.0–7.5, dissolved oxygen levels > 6.0 mg/L, and a light/dark cycle of 14:10 h. The fish were fed powdered tropical fish flakes twice daily to ensure adequate nutrition.

Non-leaching fluorescent fibrous polyester (PES) MPs were used for exposure. The PES fibers were first cut into clustered fibers and further reduced to microfibers using sterilized scissors. The microfibers were stored in sterilized glass bottles. The average length of the MPs was determined to be 300 ± 192 µm based on microscopic imaging analyzed using ImageJ software, version 1.53c (National Institutes of Health, Bethesda, MD, USA). On Day 1, zebrafish were exposed to MPs at an initial concentration of 1000 MPs/L, as described in our previous study [18]. This elevated concentration level was selected to represent a predicted future increase in global MP levels [69,75] and is attainable in environmental accumulation hotspots such as urbanized rivers. The concentration of MPs in water was monitored bi-daily and consistently maintained between 88 and 108% of the initial concentration to ensure stable exposure throughout this study.

The zebrafish in the experimental group (*n* = 36) were randomly assigned to two exposure duration groups (3 days and 7 days, 18 zebrafish each). For each exposure duration group, three replicate tanks were set up, each housing six zebrafish (a total of 18 zebrafish per group). The experimental tanks (21.7 cm × 14.6 cm) were filled with 4 L of aerated artificial freshwater, with continuous aeration maintained for 24 h to ensure adequate oxygen levels and uniform distribution of MPs. The zebrafish in the 3-day group were harvested at 72 h post-exposure, while those in the 7-day group were collected after 168 h of continuous exposure. The control group of 36 zebrafish was also maintained under identical conditions but without exposure to MPs.

At the designated sampling points (Day 3 and 7), zebrafish were euthanized on ice to minimize stress and preserve tissue integrity. Each fish was rinsed twice with Milli-Q water at room temperature to remove any externally attached MPs. Based on previous studies (Table 1) and our previous results [18], the gastrointestinal tract is the identified key organ for MP retention. Accordingly, the gastrointestinal tract, spanning from the oropharynx to the hindgut, was carefully excised under sterile conditions using autoclaved tools. For histological analysis, gastrointestinal tract samples from one fish per replicate tank (*n* = 3) in the 7-day group were collected and fixed in 30 mL of freshly prepared 50% ethanol solution. For DNA (microbiota) and RNA (transcriptomics) extraction, gastrointestinal tract samples from one fish per replicate tank (*n* = 3), for a total of 12 samples, were immediately stored at −80 °C for further analysis. Similarly, gastrointestinal tract samples from the control group were collected and processed in the same manner.

### 4.2. Gut Tissue Staining and Quantification of Mucus Secretion

For histopathological analysis, intestinal tissues from three fish per group (one fish per replicate tank, *n* = 3) in both the 7-day experimental and control groups were immersion-fixed in 10% neutral buffered formalin for 24 h to maintain architectural integrity and cytohistological morphology. Following fixation, tissues underwent sequential dehydration in a graded ethanol series, clearing in xylene, and embedding in paraffin wax. Sections with a thickness of 4 μm were prepared using a rotary microtome.

To evaluate general intestinal morphology, tissue sections were stained with hematoxylin and eosin to visualize overall tissue structure indicative of epithelial integrity and inflammatory cell infiltration. To identify acidic mucopolysaccharides, tissue sections were also stained with Alcian Blue-Periodic Acid Schiff. Stained sections were then examined under a light microscope, and representative images were captured using a digital camera system.

The mucus coverage in intestinal tissue sections was quantified using Image-Pro Plus 6.0 software’s image analyzer tool to measure the pixel count of both the stained mucus area and the total intestinal area. The mucus coverage ratio in intestinal tissue sections was quantified as the percentage of mucous area (Alcian Blue-positive regions) relative to the total area of the section. Statistical comparisons between the control and treatment groups were conducted using independent *t*-tests. Results were expressed as mean ± standard deviation, and a *p*-value < 0.05 was considered statistically significant. Slides were sealed with a mounting medium to preserve the samples and stored at room temperature for long-term analysis.

### 4.3. Gut Transcriptome Analysis

A total of 12 zebrafish (one fish per replicate tank, 3 per group) from each group were selected for mRNA sequencing analysis. Total RNA was extracted from zebrafish intestinal tissue using TRIzol reagent (Takara Bio, Dalian, China), following the manufacturer’s protocol. RNA concentration was measured using a NanoDrop 1000 spectrophotometer (Thermo Scientific, Waltham, MA, USA), and RNA integrity was assessed by the RNA Nano 6000 Assay Kit on an Agilent Bioanalyzer 2100 system (Agilent Technologies, Wilmington, DE, USA). For mRNA library preparation, poly-A mRNA was enriched using magnetic beads attached to poly-T oligos with the NEBNext Ultra RNA Library Prep Kit for Illumina (New England Biolabs, Ipswich, MA, USA). The enriched mRNA was fragmented, and first-strand cDNA was synthesized using random hexamer primers. Second-strand cDNA synthesis was performed using dTTP in non-directional library preparation. Library quality was assessed using a Qubit fluorometer (Thermo Scientific, Waltham, MA, USA), real-time PCR for quantification, and an Agilent Bioanalyzer (Agilent Technologies, Wilmington, DE, USA) for size distribution analysis. Libraries were sequenced on an Illumina NovaSeq 6000 platform (Illumina, San Diego, CA, USA) with paired-end (PE150) reads, generating approximately 6 GB of raw data per sample. Reads in FASTQ format were filtered with fastp to remove adapter sequences, reads with N bases, and low-quality reads (average Phred score < Q20). Quality metrics, including Q20, Q30, and GC content, were calculated for the clean data. High-quality reads were aligned to the zebrafish genome (version GRCz11) using HISAT2 (v2.0.5).

Differential gene expression analysis was conducted using the DESeq2 R package (v1.20.0). Genes with a Benjamini–Hochberg-adjusted *p*-value controlling the false discovery rate ≤ 0.05 were classified as differentially expressed genes (DEGs). Volcano plots were used to visually represent the DEGs, with thresholds defined as |log2FoldChange| > 1 and an adjusted *p*-value ≤ 0.05 to distinguish significantly upregulated and downregulated genes. Gene Ontology (GO) and Kyoto Encyclopedia of Genomes and Genomes (KEGG) pathway enrichment analyses were performed on DEGs to explore their biological functions and metabolic pathway involvement using the clusterProfiler package (v3.8.1), and genes were separated into up- and downregulated genes separately [80].

### 4.4. Gut Microbiota 16S rRNA Gene Sequencing Analysis

Fish gastrointestinal microbiota was sampled from a total of 12 adult zebrafish (one fish per replicate tank, 3 from each group) for 16S rRNA sequencing analysis. The 16S rRNA V4 region was PCR amplified using specific primers (515F: GTGCCAGCMGCCGCGGTAA and 806R: GGACTACNNGGGTATCTAAT) with unique barcodes for each sample. All PCR reactions were carried out with Phusion^®^ High-Fidelity PCR Master Mix (New England Biolabs, Ipswich, MA, USA). The PCR product was separated by electrophoresis on a 2% agarose gel for verification and purified with GeneJET^TM^ Gel Extraction Kit (Thermo Scientific, Waltham, MA, USA). Equal amounts of purified PCR products from each sample were pooled and subjected to end-repair, A-tailing, and ligation with Illumina adapters. Library quality was assessed using a Qubit fluorometer (Thermo Scientific, Waltham, MA, USA), real-time PCR for quantification, and a bioanalyzer for size distribution. The libraries were sequenced on the Illumina NovaSeq 6000 platform (Illumina, San Diego, CA, USA) using paired-end (PE250) sequencing, generating approximately 50,000 raw reads per library.

Paired-end reads were assigned based on their unique barcodes and were truncated by cutting off the barcodes and primer sequences. Paired-end reads were merged using FLASH (Version 1.2.11) [81]. Quality filtering of the raw reads was performed using fastp (Version 0.20.0) to obtain high-quality reads, which were then removed of chimeric sequences to obtain effective reads [82]. Effective reads were subsequently denoised using QIIME2 (2022.11) [83], following the DADA2 [84] workflow, to obtain initial amplicon sequence variants (ASVs). ASVs with an abundance of less than 5 were filtered out. Alpha diversity indices (Chao1, Simpson) were calculated in QIIME2, and sequencing depth was verified using alpha rarefaction curves to ensure comparability of all samples before inclusion and downstream analysis (Figure S4). Beta diversity was evaluated using a non-metric multidimensional scaling (NMDS) plot based on weighted UniFrac distance matrices; significant differences between groups were assessed using PERMANOVA and Adonis test. Taxonomy annotation was performed using QIIME2 (2022.11) with the SILVA v138 database [85]. PICRUSt2 was used to predictively annotate the zebrafish gut microbial functions based on 16S rRNA metabarcoding sequences [86] using the KEGG and MetaCyc databases and produce stratified tables of KEGG orthologs (KOs) and MetaCyc pathway abundances. Volcano plots were used to visualize differential abundances. Hierarchical clustering was conducted using the UPGMA algorithm. Linear discriminant analysis (LDA) effect size (LEfSe) [87] and ALDEx2 analysis [88] were conducted to complementarily identify potential differentially abundant taxa in the gut microbiota between the treatment and control groups. In the ALDEx2 analysis, taxa were evaluated using the non-parametric Wilcoxon rank-sum test on centered log-ratio (CLR) transformed abundances with 128 Monte-Carlo instances using a Benjamini–Hochberg correction threshold of <0.05; only taxa with an ALDEx2 effect size of >1.5 were considered significantly higher abundance and shown. For LEfSe, only taxa with an LDA score > 3.0 were considered significantly different and shown.

The occurrence of harmful bacteria relevant to fish was analyzed by screening the taxonomically assigned ASVs using the aquaculture bacterial pathogen database [89]. ASVs were screened at the species level, where available, after nomenclature curation. ASVs that could not be classified beyond the family level or were uncertain if they belonged to the intended genus were subsequently omitted before being subjected to downstream analysis.

## 5. Conclusions

MPs are now ubiquitously detected in nearly all aquatic environments and organisms ranging from plankton to mammals. In this study, we found that acute exposure to above environmental concentrations of polyester induced stress, which dysregulated energy metabolism, gut epithelial maintenance, and other metabolic and defense-related processes in the gut of adult zebrafish. During this process, opportunistic bacterial taxa such as *Edwardsiella* were found to significantly increase during initial exposure; microbial antioxidant functions were also potentially selected. As overall gut microbial structural balance was not significantly disrupted or had stabilized by day 7, gut microbiota showed limited compositional disruption during acute MP exposure, but the consequences of long-term indirect impacts on gut microbiota and intestinal health and homeostasis need to be empirically validated. Our findings provide new insights into the interactions between MPs, gut microbiota, and host responses, emphasizing the need for further studies to incorporate chronic exposure and functional and behavioral health impacts to better understand the broader ecological and health implications of MP pollutants.

## Figures and Tables

**Table 1:** Representative field studies reporting significant microplastic accumulation in fish gastrointestinal tracts.

Host Organism	Study Site	Major Types of MP Reported	Tissue with Highest MP Accumulation	Reference
7 riverine species (*Alburnus chalcoides*, *Barbus capito*, *Capoeta damascina*, etc.)	Qarasu River, Iran	PA, PE, PP, PS	Gut, 8.12 ± 4.26 items/individual	Makhdoumi et al., 2021 [21]
6 freshwater species (*Cyprinus carpio*, *Carassius cuvieri*, *Lepomis macrochirus*, etc.)	Han River, South Korea	PTFE, PE, rayon	Intestines, 22.0 ± 14.6 items/individual	Park et al., 2020 [76]
17 estuarine coastal species (*Nemipterus japonicas*, *Carangoides malabaricus*, *Oreochromis niloticus*, etc.)	Ennore Creek, Adyar River mouth, South India	PE, PP, PA, PS	Gut, 87% of total MPs ranging from 3.2 to 7.6 items/individual	Harikrishnan et al., 2023 [19]
13 coastal fish species (*Coilia nasus*, *Collichthys lucidus*, *Boleophthalmus pectinirostrisat*, etc.)	Hangzhou Bay and Yangtze Estuary, China	PES, PP, PE	Gut, 0.1 to 8.8 items/g	Su et al., 2019 [77]
12 coastal marine fish species (*Gastrophysus spadiceus*, *Siganus canaliculatus*, *Trachiocephalus myops*, etc.)	Beibu Gulf, China	PES, nylon, PP, PE, acrylics	Stomach, 1.25 ± 0.42 items/individual	Koongolla et al., 2020 [78]
Hybrid grouper (*Epinephelus fuscoguttatus* × *Epinephelus lanceolatus*)	Guishan Island, Zhuhai, China	HDPE, PS, ABS, PC, PTEF	Intestines, 23.91 ± 20.20 items/individual; 1.10 ± 0.86 items/g wet weight	Lam et al., 2022 [20]
dolphinfish (*Coryphaena hippurus*)	Eastern Pacific Ocean	PES, PET, PP, PS, PE-PP	Digestive tract (esophagus/stomach/intestinal tract; 0.0148–0.0560 mean items/g), 86% of total MPs	Li et al., 2022 [79]

## Data Availability

The original contributions presented in this study are included in the article. Further inquiries can be directed to the corresponding author.

## Supplementary Materials

The following supporting information can be downloaded at: https://www.mdpi.com/article/10.3390/ijms27125355/s1.
