# Supplementary material for: Gut-Microbial Responses to Acute Polyester Microplastic Exposure in Zebrafish: Dysbiosis, Opportunistic Bacteria, and Functional Impact
Source: Int J Mol Sci. 2026 Jun 13;27(12):5355. doi: 10.3390/ijms27125355 (PMC13300149; doi:10.3390/ijms27125355)
Supplement: Supplementary file 1 [file ijms-27-05355-s001.zip › ijms-4335879-supplementary.pdf]

Gut-Microbial Responses to Acute Polyester Microplastic Exposure in Zebrafish: Dysbiosis, Opportunistic Bacteria, and Functional Impact

Linus S. H. Lo [1], Liyuan Qiang [2], Peiyuan Ye [1], Cuizhu Ma [3], Keng Po Lai [4], Huahong Shi [3] and Jinping Cheng [1,*]

[1] Department of Science and Environmental Studies and State Key Laboratory of Marine Environmental Health, The Education University of Hong Kong, New Territories, Hong Kong, China
[2] College of Mechanical and Electrical Engineering, Shihezi University, Shihezi 832000, China
[3] State Key Laboratory of Estuarine and Coastal Research, East China Normal University, Shanghai 200241, China
[4] Department of Applied Science, School of Science and Technology, Hong Kong Metropolitan University, Hong Kong, China
* Correspondence: jincheng@eduhk.hk; Tel.: +(852)-2948-7881; Fax: +(852)-2948-7676

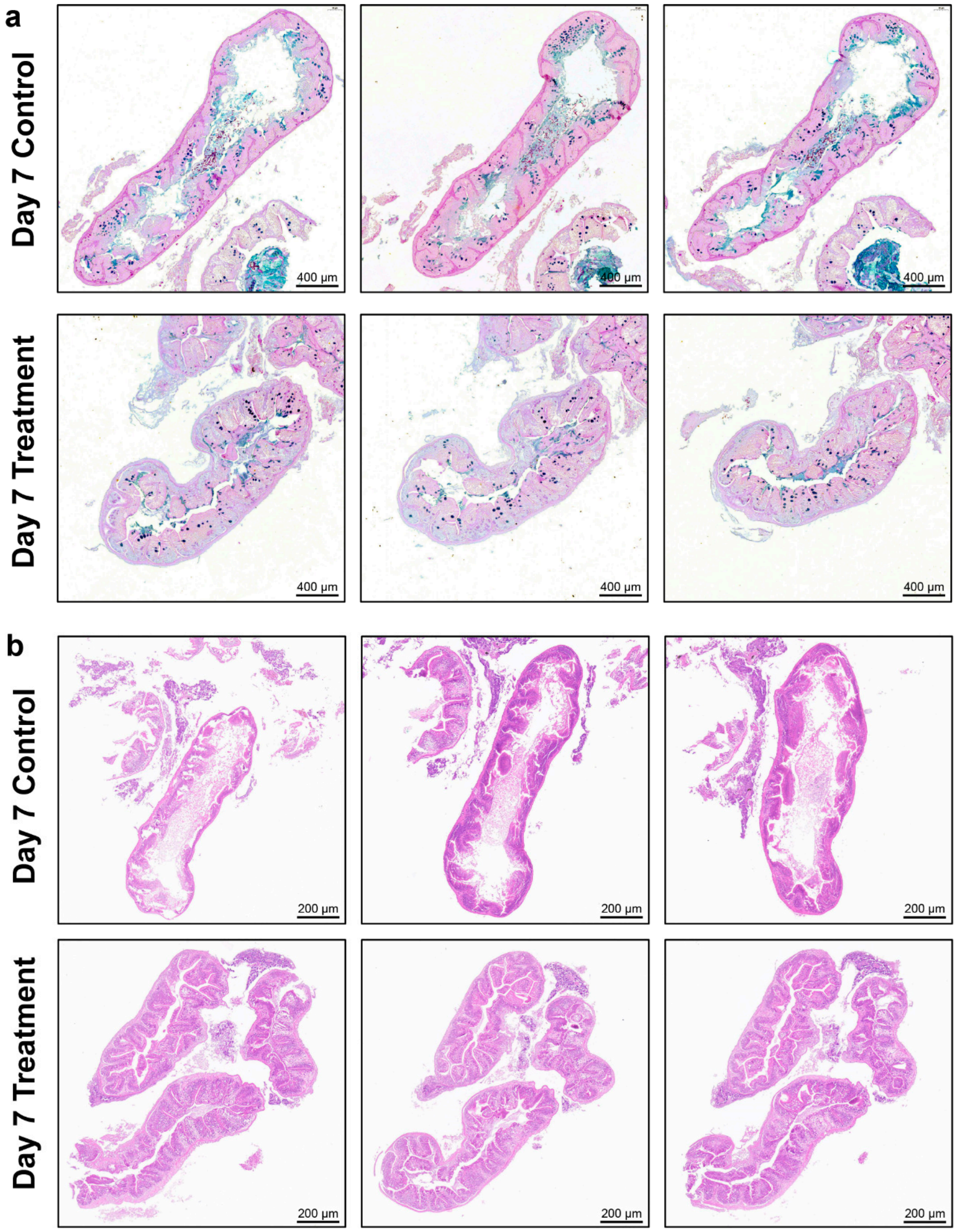

Supplementary Figure S1. Supplementary transverse sections of zebrafish gut after 7 days of microplastic exposure at 1000 items/L concentration. (a) Alcian Blue-Periodic Acid Schiff (AB-PAS) and (b) Hematoxylin and eosin (H&E) staining. Images were captured at 80× magnification.

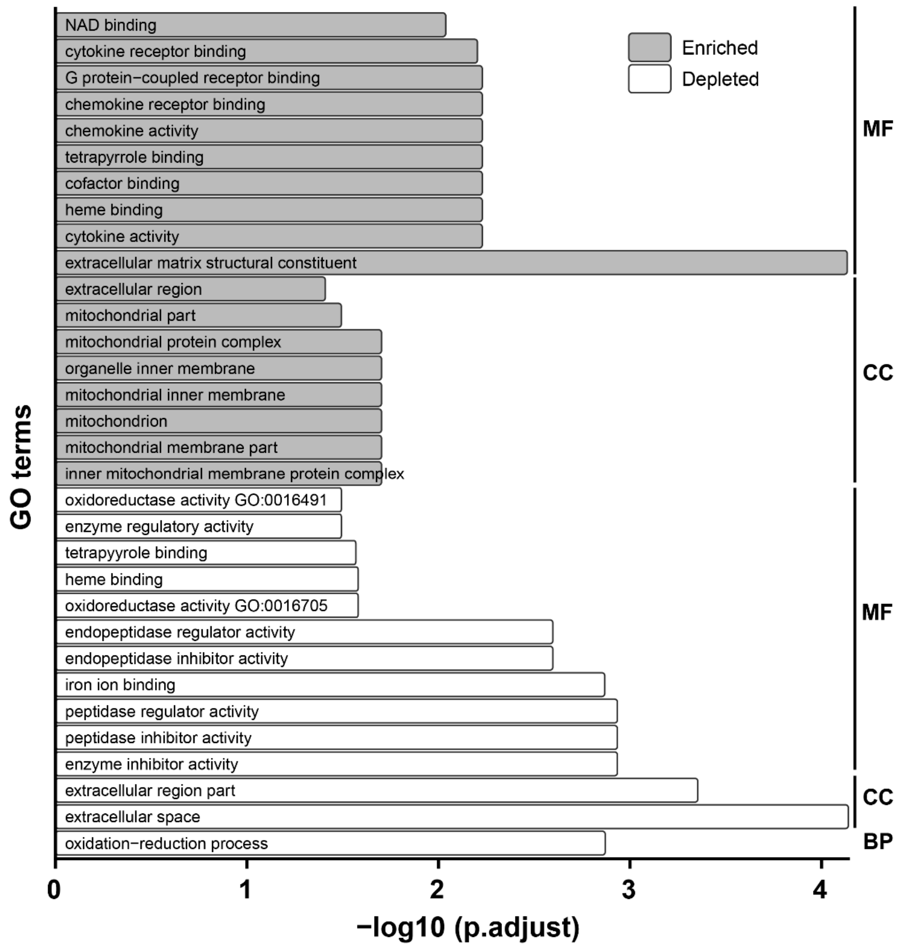

Supplementary Figure S2. GO terms enriched and depleted by upregulated and downregulated DEGs on Day 3. Only the top 10 significantly enriched terms from each of BP, CC, and MF categories are shown. P.adjust represents the Benjamini-Hochberg-adjusted P-value controlling the false discovery rate ≤ 0.05. BP: Biological Processes; CC: Cellular Components; MF: Molecular Function.

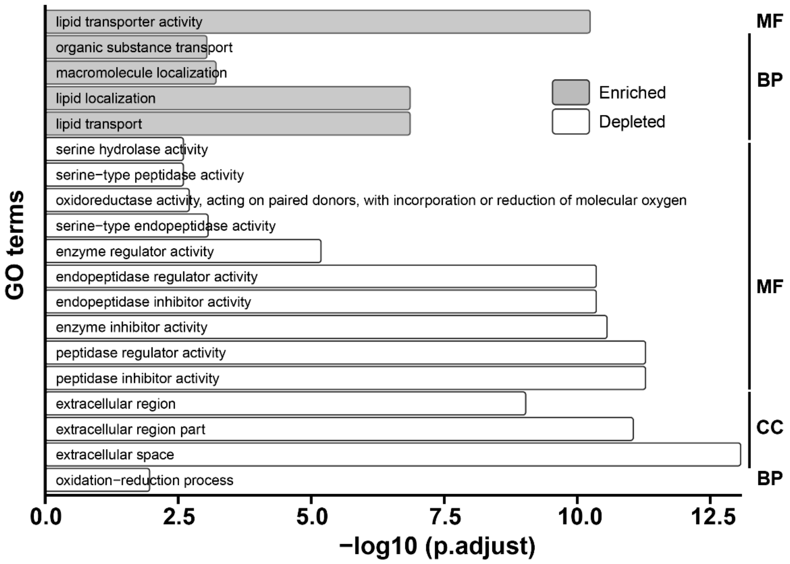

Supplementary Figure S3. GO terms enriched and depleted by upregulated and downregulated DEGs on Day 7. Only the top 10 significantly enriched terms from each of BP, CC, and MF categories are shown. P.adjust represents the Benjamini-Hochberg-adjusted P-value controlling the false discovery rate ≤ 0.05. BP: Biological Processes; CC: Cellular Components; MF: Molecular Function.

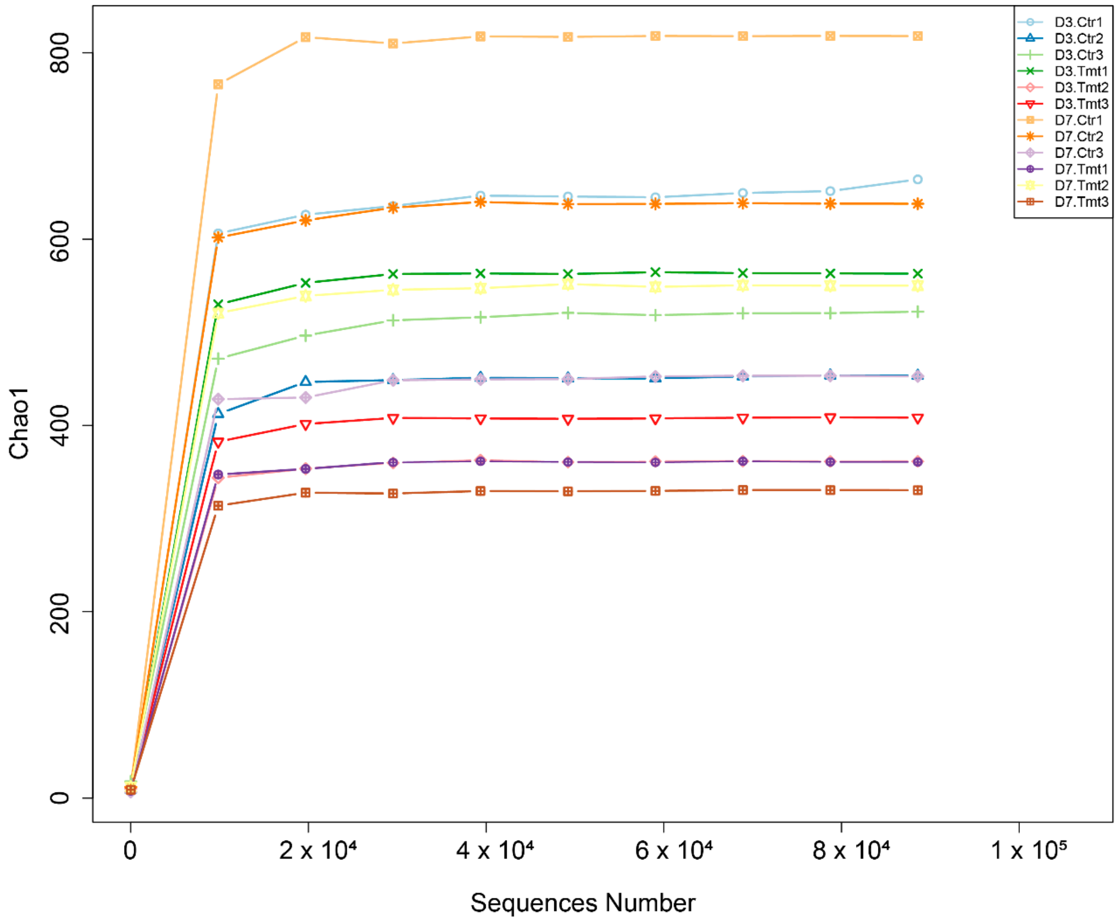

Supplementary Figure S4. Rarefaction curves showing the change in alpha diversity of each sample with sequencing depth using the Chao1 index. D3: Day 3; D7: Day 7; Ctr: Control; Tmt: Treatment.
